# Prevalence, characteristics and risk factors of imported and local malaria cases in North-Western Province, Zambia: a cross-sectional study

**DOI:** 10.1186/s12936-020-03504-1

**Published:** 2020-11-23

**Authors:** Maureen N. Chipoya, Nzooma M. Shimaponda-Mataa

**Affiliations:** Department of Biomedical Sciences, School of Health Sciences, University of Zambia, Ridgeway Campus, Lusaka, Zambia

**Keywords:** Local malaria, Imported malaria, Risk factors, Gametocytaemia, Zambia

## Abstract

**Background:**

Imported malaria is a major challenge for countries that are in malaria elimination stage such as Zambia. Legitimate cross-border activities add to the risk of transmission, necessitating determination of prevalence, characteristics and risk factors of imported and local malaria.

**Methods:**

This cross-sectional study was conducted in 103 consented child and adult patients with clinical malaria symptoms, from selected health facilities in north-western Zambia. Patient demographic data and blood samples for malaria microscopy and full blood count were obtained. Chi-square and penalized logistic regression were performed to describe the characteristics and assess the risk factors of imported and local malaria in North-Western Province.

**Results:**

Overall, malaria prevalence was 78.6% with 93.8% *Plasmodium falciparum* and 6.2% other species. The local cases were 72 (88.9%) while the imported were 9 (11.1%) out of the 81 positive participants. About 98.6% of the local cases were *P. falciparum* compared to 55.6% (χ^2^ = 52.4; p < 0.01) *P. falciparum* among the imported cases. Among the imported cases, 44% were species other than *P. falciparum* (χ^2^ = 48; p < 0.01) while among the local cases only 1.4% were. Gametocytes were present in 44% of the imported malaria cases and only in 2.8% of the local cases (χ^2^ = 48; p < 0.01). About 48.6% of local participants had severe anaemia compared to 33.3% of participants from the two neighbouring countries who had (χ^2^ = 4.9; p = 0.03). In the final model, only country of residence related positively to presence of species other than *P. falciparum* (OR = 39.0, CI [5.9, 445.9]; p < 0.01) and presence of gametocytes (OR = 23.1, CI [4.2, 161.6]; p < 0.01).

**Conclusion:**

Malaria prevalence in North-Western Province is high, with *P. falciparum* as the predominant species although importation of *Plasmodium ovale* and *Plasmodium malariae* is happening as well. Country of residence of patients is a major risk factor for malaria species and gametocyte presence. The need for enhanced malaria control with specific focus on border controls to detect and treat, for specific diagnosis and treatment according to species obtaining, for further research in the role of species and gametocytaemia in imported malaria, cannot be overemphasized.

## Background

Malaria continues to be among the most prevalent parasitic diseases experienced worldwide [1]. Globally, an estimated 228 million malaria cases and 405,000 deaths were recorded in 2018 compared to 231 million cases and 416,000 deaths in 2017 [2]. The majority of this burden rests on sub-Saharan Africa and India, which carry almost 85% of it [2]. In the African Region, malaria accounts for about 93% of the cases [2]. In Zambia, malaria is equally of concern as it is a major cause of mortality and morbidity [3] in highly endemic areas [4–6]. As of 2018, the Ministry of Health reported presumed and confirmed cases of up to 5,193,723 and deaths of up to 1209 [2].

The malaria burden in Zambia is greatly influenced by climatic factors, such as rainfall and temperature [7]. Rainfall mainly contributes to the availability of breeding habitat for mosquito vectors [8]. On the other hand, temperature determines the length of development of larval mosquitoes and malaria parasites, as well as the lifespan and the rate of blood feeding of adult vectors [8]. The burden of malaria continues to be highest in Northern, Luapula and Muchinga provinces, which fall in the high malaria transmission zone [6]. These are followed by other provinces including the North-Western Province [9, 10], which is officially known to belong to the medium malaria transmission zone [6]. North-Western Province has high rainfall, many rivers, thick forestry, dams and swamps. Further, the major mining activities in the province allow for various occupational activities, leading to high population [11]. The suitable environmental conditions would speak to the generally high malaria cases [7, 8].

Focussing more on North-Western Province, this is a province bordered by two countries, the Democratic Republic of Congo and Angola [11]. This geographic location may contribute to malaria importation given that malaria parasites do not respect national borders and are exacerbated by human mobility [12]. The Democratic Republic of Congo and Angola were among the six leading contributors to the 85% global malaria mortality burden that was concentrated in 20 countries in the African region and India in 2018 [2]. In 2018, the African sub-region had about 49 million estimated cases representing a 7% increase to which the Democratic Republic of Congo and Angola were the leading contributors by approximately 55% and 14%, respectively [2].

Among the five species of malaria, *Plasmodium falciparum* accounted for 99.7% of morbidity in the World Health Organization (WHO) African Region in 2018 [2]. Although the WHO shows only 2% *Plasmodium vivax* and 98% *P. falciparum* in Africa, and only *P. falciparum* in southern Africa [2], Sitali and others showed a *Plasmodium* species distribution of 88.4% *P. falciparum*, 0.8% *Plasmodium malariae* and 0.4% *Plasmodium ovale* in Luapula and Eastern provinces of Zambia [13]. *Plasmodium vivax* was also reported but only as mixed infections with other species and these were distributed as 0.2% *P. falciparum* with *P. vivax* and 0.2% *P. falciparum* with *P. malariae* and *P. vivax* [13]. The rest were mixed infections of 6.5% *P. falciparum* with *P. malariae*, 2.1% *P. falciparum* with *P. ovale* and 1.3% *P. falciparum* with *P. malariae* and *P. ovale* [13]. Zhou et al. also showed importation of *P. falciparum, P. ovale* and *P. vivax* to China from 20 African countries, with Angola, Congo, Equatorial Guinea, Nigeria, and Liberia being the top five sources of *P. ovale* and only Angola, Equatorial Guinea, Congo, and Guinea being sources of *P. vivax* [14]. Additionally, according to unpublished hospital records from some health facilities in North-Western Province, the species being seen in the province may include species other than *P. falciparum.* Considering that most Government Health Facilities use the *P. falciparum* specific rapid diagnostic tests (RDTs), such species are likely to be missed in some health facilities that have no laboratory capacity to detect other species.

Reducing the incidence of malaria is a national priority that requires a focused comprehensive and consistent approach in order to achieve the vision of “a malaria free-Zambia by 2030” [15]. So far, the National Malaria Elimination Centre (NMEC) efforts have contributed to a 40% reduction of deaths and a promising 40% reduction in cases by 2020 [2]. Although Zambia is not among the 21 countries that are part of the WHO “E-2020 initiative” identified by the WHO to work towards the 2020 elimination milestone of the global malaria strategy [2], it is a member of the Elimination Eight (E8) Regional Initiative, a similar elimination initiative at regional level in Southern Africa [16]. This initiative includes eight countries and works in a phased approach to eliminate malaria initially in four frontline countries i.e. Botswana, Namibia, South Africa and Swaziland by 2020, and later pave the way for elimination in four second-line countries (i.e. Angola, Mozambique, Zambia and Zimbabwe) by 2030 [16]. In supporting the four frontline countries, Zambia and the other three second-line countries are working to scale up malaria control, logistically, strategically and politically especially in border areas, in terms of a series of cross-border initiatives [16]. This is because increasing travel to endemic areas in recent decades contributes to imported malaria cases, which continue to pose challenges for diagnosis and management [16]. This is especially so, given the fact that currently, criteria on definitions of time periods qualifying a case to be termed an import, are not clear or standardized [17]. The criteria vary from 18 days to 2 months among countries and 3 months by the WHO [17]. Data on the features of imported cases can provide valuable information about both the epidemiology of malaria in endemic regions where surveillance systems are weak [12] as well as responsible factors in non-endemic regions [18]. The movement of malaria in endemic countries has also contributed to the spread of drug resistance and threatens long-term eradication goals [15, 19].

Control and treatment strategies in countries like Zambia, that are in the elimination stage should account for the continued globalization of malaria and increase efforts to understand patterns of human and malaria parasite movement [8, 12]. Further, how such information can guide control and elimination strategies especially given the increasing focus on malaria importation and the vulnerability of countries to resurgence should also be prioritized [8, 12]. As such, studying the characteristics of the local and imported malaria cases prevailing will assist in the development of more effective future malaria surveillance and prevention programmes [8], accurate detection and diagnosis [20], streamlined treatment depending on species and the varying severity of malaria and in educating the community in adherence to preventive measures. This study, therefore, sought to evaluate the characteristics of imported and local malaria cases in North-Western Province, Zambia. It further sought to determine the prevalence of malaria and highlight risk factors that could be considered in targeted prevention and control strategies.

## Methods

### Study design

A health facility based, cross sectional study was conducted from March to September 2019. Participants were drawn from patients of all age groups from 3 months old, with signs and symptoms of malaria and seeking medical attention from the three selected health facilities of North-Western province, Zambia.

### Study setting

The study was conducted in North-Western Province, one of the ten Provinces of Zambia. This province covers an area of 125,826 km^2^, has a population of 727,044 and a population density of 5.80 per km^2^ [21]. It is one of the sparsely populated provinces in the country. The province is divided into ten districts namely, Chavuma, Ikelenge, Kabompo, Kasempa, Manyinga, Mufumbwe, Mwinilunga, Solwezi, Mushindamo and Zambezi. The rural population constitutes 77.5%, while the urban population makes up the remaining 22.5%. North-Western Province is bordered by Angola in the west, the Democratic Republic of Congo (Congo D. R.) in the north, Copperbelt Province in the southeast, Central Province in the south, Western Province in the west [11]. The major activities that take place within the province are farming, fishing, trading and mining. The study sampled from three health centres nearest to Congo and Angola namely; Jimbe Rural Health Centre, 2 km away from Angola and about 390 km away from Solwezi District; Sachibondu Rural Health Centre, 16 km away from Congo and about 321 km away from Solwezi District.; and Mushindamo Rural Health Centre, in Mushindano District, 15 km away from Congo and about 137 km from Solwezi District.

Both Jimbe and Sachibondu Rural Health Centres are in Ikelenge District which is located 340 km from Solwezi District. Jimbe Rural Health Centre has an in-patient capacity of five beds, and covers a population of 3384 people. Sachibondu Rural Health Centre has an in-patient capacity of 25 beds. It covers a population of about 5000 people. Mushindano Rural Health Centre has an in-patient capacity of 16 beds and covers a population of about 9200 people.

Jimbe and Mushindano Rural Health Centres have no Laboratories and referral cases are mostly sent to Solwezi General Hospital.

### Inclusion and exclusion criteria

Inclusion criteria for the study were: attendance at any of the three selected health facilities, of all age groups starting from 3 months old, with signs and symptoms of malaria, residence in North-Western Province and no history of travel 4 weeks prior to attending the health facility, residence in Angola or Congo with no history of travel 4 weeks prior to attending the health facility apart from travel to health facility which should have taken place within 5 to 7 days of leaving their residence.

Patients were excluded from the study if they: were under 3 months of age, were extremely ill and in need of urgent medical attention at the time of testing, had any known complications from diseases other than malaria, had taken any anti-malarial drug in the previous 3 weeks.

Travel history and anti-malarial drug data were obtained via open-ended questions asking specifically when they had last travelled out of their district or crossed borders or taken anti-malaria drugs. This was done in order to mask the intent of the questions and reduce bias. The study also used calendars to minimize recall bias by allowing the recall process to be as independent as possible.

### Sample size determination and enrolment

An estimated sample size of 103 was obtained based on the 6% prevalence of malaria in the medium malaria transmission zone where North-Western Province falls [22]; by sample size formula for descriptive studies, N = Z^2^p (100-p)/𝓮^2^; where N (87) was calculated as the sample required; Z, the Standard error (1.96); e, Precision required (95%, 0.05) and p, Estimated prevalence (6%) [22]; and after factoring in non-response rate of 15%, implying a response rate of 85%; the adjusted sample size was calculated as 87/(1–0.15) = 102.7; Sample size (n) = 103. As such, a total of 103 consented and qualifying patients, were enrolled by convenience sampling. These were broken down as follows: Jimbe—34 patients; Sachibondu—34 patients; and Mushindamo—35 patients.

### Data collection

All data collection was performed using standard recruitment and testing protocols by specific trained study staff to reduce bias. After confirming that the patients had not travelled and had not taken any anti-malarial drug as per inclusion and exclusion criteria, patients were taken through the informed consent (and or assent). This process was to furnish them with information about the study and request them to voluntarily participate in the study. Adult patients were requested to sign or fingerprint on the consent form while for child patients, parents or guardians assented by the same token.

For all consented (assented) patients, clinical data including demographic details of the patients, risk factors, and presenting signs and symptoms were collected from medical records of the patients after clinical examinations by the Health Professionals Council of Zambia registered Clinicians in the facilities; and through semi-structured interviews with patients, parents or guardians by the study team. The interviews, study information sheet and written informed consent were developed in English but administered to the patients in a specific local language the patients or guardians were comfortable with among the four main languages namely; Lunda, Luvale, Kaonde and Lamba spoken in North-Western Province, and Lunda and Swahili for participants from Angola and Congo, respectively.

Finger prick blood specimens for RDTs and venous blood in EDTA containers for thick and thin blood smears as well as full blood count (FBC) determination, were obtained from the recruited patients.

### Description of laboratory procedures

The SD BIOLINE Malaria Ag P.f POCT, a rapid qualitative diagnostic test manufactured by Standard Diagnostics, Inc., used for the detection of histidine-rich protein II (HRP-II), a malaria antigen specific to *P. falciparum* in human blood specimens, was employed. This was mainly for initial determination of *P. falciparum* positive individuals to facilitate immediate treatment needs. *Plasmodium falciparum* positive result was confirmed by the appearance of two red/pink lines in the test area of the RDT cassette representing the test and control reactions while a negative result was confirmed by the appearance of only one red/pink control line. This process was conducted for all the 103 participants enrolled and results were recorded as SD BIOLINE Malaria Ag P.f POCT RDT positive or negative.

From the venous blood specimens collected, both thick and thin films were prepared and the haemoglobin (HB) levels determined. For the samples collected from Jimbe and Sachibondu Health centres, HB determination and smear preparations were done at Mwinilunga General Hospital by a laboratory technician, while for those collected from Mushindamo Health centre, both procedures were performed at Solwezi General Hospital by laboratory technicians stationed there.

All slides from the three health facilities were read at Solwezi General Hospital by the principal investigator as the first reader. The second and third (blind) slide readers were a Medical Laboratory Technologist and a Biomedical Scientist to confirm species and parasitaemia for quality control purposes, to reduce bias.

### Determination of parasitaemia and species by microscopy

Thin blood smears were air dried, fixed with 95% methanol for 1 min and then stained in three percent Giemsa for 45 min, while thick blood smears were dehaemoglobinized in buffer for 30–60 s for transparency of the red blood cells then stained with ten percent Giemsa at pH 7.2–7.4 for 15 min. The stained smears were examined under light microscopy at 100× magnification. Thick blood smears were used for detection and quantification both asexual and sexual forms of malaria parasites. Thin blood smears were used for species identification [9, 23, 24].

Peripheral parasite density calculations were based on the number of asexual forms and leukocytes or white blood cells (WBCs) counted [23] and converted into parasites per microlitre of blood based on the WHO-recommended average of 8000 WBCs per microlitre of blood in every human being [25]. Briefly, this procedure involved tallying the parasites (using one tally counter) against WBCs (using a second tally counter), until the slide reader counted 100 parasites or 200 WBCs, whichever came first. Thereafter, expressing the results as parasites per microlitre of blood, using the formula: Parasites/microlitre blood = (parasites/WBCs counted) × 8000 WBCs per microlitre blood. Where no parasites were detected, slide readers examined up to 500 WBCs or 200 microscope fields of the thick films before assigning a negative result.

### Evaluating the severity of malaria in imported and local malaria cases

Given that severe malaria is defined as either Blantyre coma score < 5 or severe anaemia < 5 g/dl when they co-exist with parasitaemia and an auxiliary temperature of > 37.5 °C and other criteria as listed in the WHO criteria for severe malaria; and uncomplicated malaria is defined as *P. falciparum* parasitaemia and an axillary temperature of < 37.5 °C, or parasitaemia and symptoms suggestive of malaria [26–28], severity of anaemia was determined based on haemoglobin levels in the participants.

HB levels were determined using full blood counts obtained from venous blood samples that were run on ABX micros 60 FBC machine. Participants who had haemoglobin levels below 5.0 g/dl [26–28] were considered as having severe anaemia, while participants with haemoglobin levels higher than 5.0 g/dl were considered to have mild or moderate anaemia.

### Determining risk factors associated with imported and local malaria cases

The following data i.e. age, gender and country of residence were analysed statistically to determine the risk factors associated with the outcomes. Data analysis staff were blinded to patient exposure and outcome status in order to reduce bias. Data entry and cleaning were done concurrently with laboratory procedures. This allowed for re-confirmation with patient records or deleting and replacing of potential participants with completely missing data.

### Statistical analysis

The data was analyzed using Microsoft Excel 2013, IBM-Statistical Package for Social Sciences (IBM-SPSS) version 23 and R version 4.02. In the univariate analysis, data were described by summarizing continuous variables such as age, into means and standard deviations and reporting categorical variables such as gender, malaria positivity, anaemia and malaria species as frequencies and percentages.

Chi-Square (χ^2^) was used for the bivariate analysis to determine relationships between categorical, independent variables i.e. country, age and gender with the outcome variables malaria positivity, malaria species, severity and gametocyte presence.

Given the following considerations (i) that species other than *P. falciparum* are uncommon in patients attending most health facilities in Zambia, (ii) that gametocyte detection by microscopy is usually low; (iii) that some outcome categories in the data set were so small and highly unbalanced, it was recognized that this would cause an underestimation of the probability of occurrence and biased standard errors [29]. Therefore, Firth logistic regression method, a type of penalized likelihood models, which is recommended for rare events and also deals with quasi-complete separation of data points to reduce bias, was used [30]. Two single-predictor Firth logistic regression models were run in R version 4.02 in the Windows 10 environment, using the “logisticf” package [31], with results interpreted the same way as in traditional logistic regressions. The models were run with presence of (a) species and (b) gametocytes as dependent, outcome variables and country, gender and age as predictors and independent variables. Both outcome and predictor variables, were coded with one (1) when the value of interest was present and with zero (0) for a value otherwise. Ultimately the two models were used to estimate country-associated risks for outcome or dependent binary variables, malaria species and gametocyte presence while controlling for confounders, such as age and gender. Odds ratios and 95% confidence intervals (CI) were reported. Statistical significance was set at a level of p < 0.05.

## Results

### Demographic characteristics

Out of the 103 participants, 43 (41.7%) were males and 60 (58.3%) females, with a mean age of 12 years [range: 0.3 (4 months)–72 years; standard deviation: 11.7]. Table 1 presents a summary of the demographic characteristics of the study population, including child/adult categories (child/paediatric: 15 years and below; adult: above 15 years), gender, country of residence and malaria positivity.

**Table 1:** Univariate analysis—demographic characteristics of patients attending selected health facilities in North-Western Province, Zambia enrolled into the study

Variables	Frequency N = 103
Age category	
Child	73 (70.9%)
Adult	30 (29.1%)
Gender	
Female	60 (58.3%)
Male	43 (41.7%)
Country	
Zambia	93 (90.3%)
Congo & Angola	10 (9.7%)
Rapid diagnostic test positivity	
Positive	88 (85.4%)
Negative	15 (14.6%)
Malaria microscopy positivity	
Positive	81 (78.6%)
Negative	22 (21.4%)

**Fig. 1:**
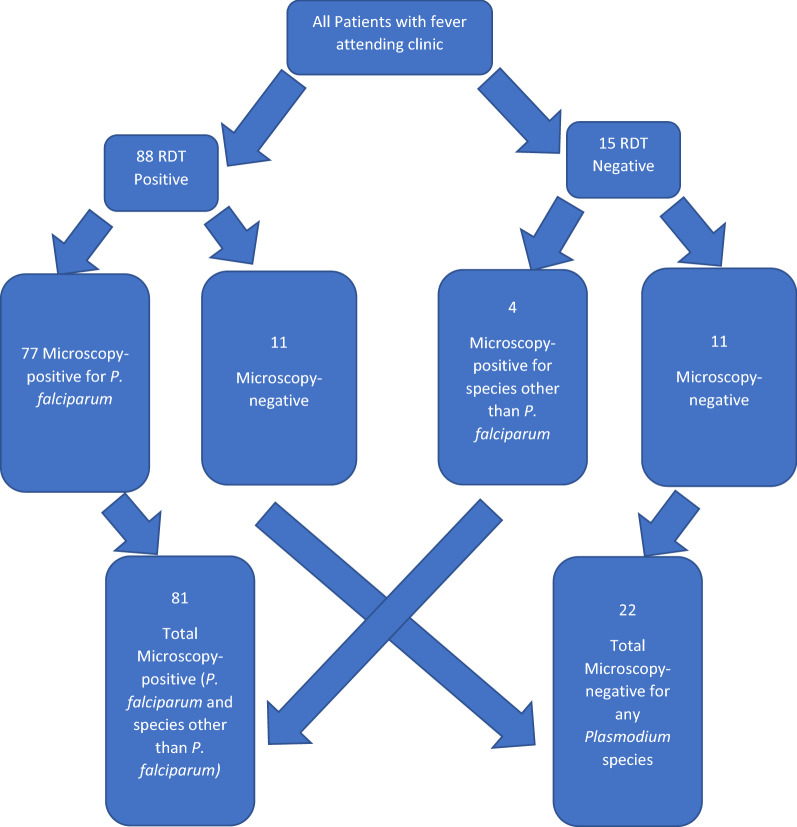
Enrollment process for participants into the study

From Table 1, a discrepancy between RDT (85.4%) and microscopy (78.6%) diagnosis was demonstrated. Out of the 88 participants who were *P. falciparum* positive by RDT, only 77 participants were *P. falciparum* positive by microscopy, rendering 11 from the RDT as false positives. On the other hand, out of the 15 RDT negative participants, four were microscopically positive for other species. Figure 1 illustrates the enrolment process and sample characteristics.

Table 2 presents the three (3) variables used to describe the characteristics of malaria in the study as presence of (1) species, (2) gametocytes and (3) severe anaemia.

**Table 2:** Bivariate analysis—risk factors for characteristics (species; gametocytes and severity) of local and imported malaria in patients attending selected health facilities in North-Western Province, Zambia enrolled into the study

Independent variables	Malaria characteristics (dependent variables)					
	Species		Gametocytes		Anaemia	
Categories	1 = Species other than *P. falciparum*	0 =* P. falciparum*	1 = Gametocytes present	0 = Gametocytes absent	1 = Severe anaemia	0 = Moderate/normal
Age group						
Children	2 (3.6%)*	53 (96.4%)	3 (5.5%)	52 (94.5%)	27 (49.1%)	28 (50.9%)
Adults	3 (11.5%)	23 (88.5%)	3 (11.5%)	23 (88.5%)	11 (42.3%)	15 (57.7%)
Gender						
Female	3 (6.1%)	46 (93.9%)	4 (8.2%)	45 (91.8%)	26 (53.1%)*	23 (46.9%)
Male	2 (6.3)	30 (93.7%)	2 (6.3%)	30 (93.8%)	12 (37.5%)	20 (62.5%)
Country						
Participants from Congo and Angola	4 (44.4%)*	5 (55.6%)	4 (44.4%)*	5 (55.6%)	3 (33.3%)*	6 (66.7%)
Participants from Zambia	1 (1.4%)	71 (98.6%)	2 (2.8%)	70 (97.2%)	35 (48.6%)	37 (51.4%)

*p < 0.05

Overall, the 81 out of 103 patients that tested positive for malaria by microscopy translated to a prevalence of 78.6%. The species found in the malaria positive participants were 76 (93.8%) *P. falciparum* and 5 (6.2%) for other species. Disaggregating the malaria cases by local and imported, 72 (88.9%) of the 81 positive participants were local cases and 9 (11.1%) were imported.

Based on the characteristics of both the local and imported malaria cases investigated, namely; species, gametocyte presence and severity of anaemia, this study shows that 71 of the 72 local cases (98.6%) were *P. falciparum* and only one (1.4%) was *P. malariae*; and five (three from Angola and two from Congo) out of nine imported cases (55.6%) were *P. falciparum* although one case was a mixed infection of *P. falciparum* and *P. malariae*. The remaining four out of the nine imported cases (44.4%) were of species other than *P. falciparum* broken down as follows: two out of nine *P. ovale* (22.2%) occurring in participants from Angola and Congo each; one out of nine (11.1%) with a mixed infection of *P. ovale* and *P. malariae*, occurring in a participant from Angola and yet another one out of nine (11.1%) with a mixed infection of *P. falciparum* and *P. malariae*, occurring in a participant from Congo.

**Fig. 2:**
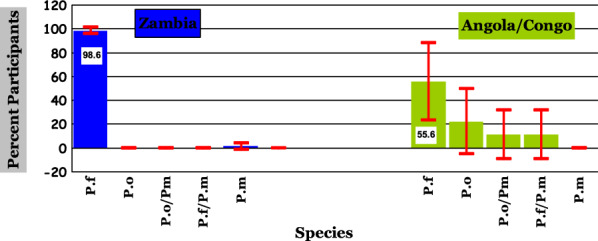
*Plasmodium* species identified in the malaria cases recruited

Figure 2 shows that the *Plasmodium* species picture in the participants resident in Zambia differs significantly from that in participants from the two neighbouring countries. Participants resident in Zambia had *P. falciparum* (98.6%: 95% CI 95.9–101.3) as the main species, while participants from the two neighbouring countries had a considerable combination of *P. falciparum* (55.6%: 95% CI 23.1–88), *P. ovale* (22.2%: 95% CI 4.9–49.4), *P. ovale/P. malariae* and *P. falciparum/P. malariae* (11.1%: 95% CI 9.4–11.1) each. Table 2 further shows that out of the five cases of species other than *P. falciparum*, three were adult cases (11.5%) and two were children (3.6%; χ^2^ = 4.5; p = 0.03), varying significantly.

With regards to the other two characteristics studied, i.e. presence of gametocytes and severe anaemia, there were four participants (44%) with gametocytes from the two neighbouring countries, which is significantly more than the two cases (2.8%; χ^2^ = 48; p < 0.001) observed in the participants resident in Zambia.

In terms of severe anaemia, Table 2 shows that this varied significantly by gender and country where 26 females (53.1%) compared to 12 males representing 37.5% (χ^2^ = 4.9; p = 0.03) and three (33.3%) from the two neighbouring countries and 35 (48.6%) from Zambia (χ^2^ = 4.8; p = 0.03) had severe anaemia.

Firth Penalized Regression models were run for the significant cases in the bivariate analysis to determine the associated risk factors. Table 3 shows that country of residence related positively to presence of species other than *P. falciparum* (OR = 39.0, CI [5.9, 445.9]; P < 0.01) as well as presence of gametocytes (OR = 23.1, CI [4.2, 161.6]; P < 0.01). Thus participants who resided in Angola and Congo were more likely to have species other than *P. falciparum* as well as gametocytes. Age and gender were not associated with either presence of species other than *P. falciparum* or gametocytes. Country, age and gender were all not significant in the severe anaemia analysis.

**Table 3:** Firth (penalized) regression analysis of risk factors for characteristics of local and imported malaria in patients attending selected health facilities in North-Western Province, Zambia, enrolled into the study

Predictor	Coef.	*SE* Coef.	Wald’s χ^2^	*df*	*p*	eβ (odds ratio)		
Species								
Constant	− 3.864	0.831	Inf	1	< 0.01	N/A		
Country (1 = Congo & Angola; 0 = Zambia)	3.664	1.067	14.7	1	< 0.01	39.0	5.9	445.9
Test overall model evaluation			χ^2^	*df*	*p*			
Likelihood ratio test			14.7	1	< 0.01			
Wald test			11.8	1	< 0.01			
Gametocytes								
Constant	− 3.339	0.648	Inf	1	< 0.01	N/A		
Country (1 = Congo & Angola; 0 = Zambia)	3.139	0.932	12.5	1	< 0.01	23.1	4.2	161.6
Test overall model evaluation			χ^2^	*df*	*p*			
Likelihood ratio test			12.5	1	< 0.01			
Wald test			11.3	1	< 0.01			

*CI* confidence interval, *Coef.* coefficient, *SE coef.* standard error of coefficient, *χ*^*2*^ Chi square; *df* degrees of freedom, *eβ* exponent of coefficient, *Inf* infinity

These results show that: Predicted logit of (species presence) = −3.864 + (3.664)*Country; and Predicted logit of (gametocyte occurrence) = −3.339 + (3.139)*Country.

According to the models, the odds of patients residing in the two neighbouring countries who attend the selected health facilities in North-Western Province, having species other than *P. falciparum* were 39.1 times (= e^3.664^; Table 3) times greater than the odds of patients residing in Zambia who attend the same selected health facilities in North-Western Province having these species; and the odds of patients residing in the two neighbouring countries who attend the selected health facilities in North-Western Province, having gametocytes were 23.1 times (= e^3.139^; Table 3) times greater than the odds of patients residing in Zambia who attend the same selected health facilities in North-Western Province, having gametocytes.

## Discussion

This current study examines the characteristics of local and imported malaria cases in selected health facilities of some border areas of north-western Zambia. An overall malaria prevalence of 78.6% was observed. These findings are slightly higher but comparable with other findings [13] based on the analysis of data from the 2012 Zambia Malaria Indicator Survey (MIS) which showed 52.6% and 55.6% malaria prevalence for Eastern and Luapula provinces of Zambia respectively [13]. The findings are consistent with another study [7], which determined that the incidence of malaria in North-Western Province did not vary from that of Luapula Province. This was in the context that although North-Western Province belonged to the moderate malaria transmission zone in Zambia, its malaria burden approximated that of high malaria transmission zones [7]. As a matter of fact, north-western Zambia generally receives quite high rainfall amounts and has environmental conditions suitable for malaria vector breeding [7, 8]. As such, malaria transmission zones are indicative of the general burden in delineated geographical locations based on general environmental and climatic conditions and yet actual burden may vary by specific places because the specific environmental and climatic conditions vary even within the same transmission zone [7].

Disaggregating the prevalence of malaria by local and imported cases, this study, demonstrated that majority (88.9%) of the malaria cases were local, as expected, although a few (11.1%) imported cases were also detected. These were of patients from the two neighbouring countries, Angola and Congo, who accessed the selected health facilities in Zambia during the period of our study. The two countries Angola and Congo have been reported to be among the leading countries in the importation of malaria from Africa to other countries, such as China [14]. With regards to imported malaria, this study discusses the findings in perspective of other national survey findings, in spite of the differences in study design and duration. Here this study employs the definition of imported malaria, that “cases are regarded as imported infections when in-migration occurred within 5–7 days of a case being detected” [32]. As such, the imported malaria prevalence in this study was lower than most surveys reviewed. The prevalence of imported malaria was higher in most studies; given the 64% in China [14], about 14 to 90% in Iran [33], 100% in South Africa [34], 53% in Swaziland [35] and 94.2% in Lusaka, Zambia [36]. However, a study conducted in Botswana showed a lower prevalence of 6.01% [37], comparable to the findings in this study.

Discussing this study conducted in a moderate malaria transmission zone, in perspective these surveys, makes it clear that imported malaria occurs in low [34, 36], high [34] as well as moderate transmission zones, in varying proportions and without a pattern specific for particular zones. For a long time now the World Health Organization (WHO), has been recommending quantifying of the importation of infections into a region [38]. This is advised in order to classify regions according to their “vulnerability” [38], and also to estimate the risk of malaria being reintroduced following elimination efforts [39]. However, the focus has been more on the role of the latter, to prevent negating the malaria elimination successes [40, 41]. Given the possibility of malaria importation occurring in all zones of malaria transmission, it is very fundamental to pay attention to imported malaria infections even when local incidence is high [42]. This is crucial because importation will contribute to enhance the parasite reservoir and affect any elimination and control successes [42]. It has been recognized that staff at borders can also contribute to transmission of imported malaria [43], another reason why paying attention to imported malaria and border control is critical. Countries that have scored successes in eliminating malaria have among other strategies, employed border controls to detect and treat malaria infections before they reach receptive areas of the countries they enter [34]. Additionally, such countries have also integrated other strategies like economic development [32] and cross-border vector control [32, 40].

This study determined the characteristics of both the local and imported malaria cases based on species, gametocyte presence and severity of anaemia. The two models, show that country of residence is related to both species and gametocyte presence. *P. falciparum* as well as *P. malariae* and *P. ovale,* referred to as “species other than *P. falciparum”* in this study, were present in patients attending the selected health facilities of north-western Zambia. A prevalence of 93.8% *P. falciparum* and 6.2% of other *Plasmodium* species was found in this study. These findings are consistent with the Zambia 2012 MIS study, which demonstrated prevalence of 88.3% *P. falciparum* species and 11.6% non-falciparum species [13]. Like the current study, the MIS study [13] also demonstrated that *Plasmodium* species other than the main *Plasmodium falciparum* were present, especially in areas that have proximity to border areas [13]. The Zambia Ministry of Health publications also indicate that species other than *Plasmodium falciparum* are present in Zambia, in low proportions [28]. According to the Zambia Ministry of Health, *Plasmodium falciparum* accounts for more than 95%, while *P. malariae* makes up 3% and *P. ovale* 2% [28].

In this study, the species other than *P. falciparum*, were missed by the initial RDT screening and were only detected by microscopy. This is attributed to the *P. falciparum*-specific RDTs employed. As a matter of fact, the *P. falciparum*-specific RDTs are the only kits available in most health facilities, as per recommendation and provision by the Ministry of Health [28]. This recommendation was effected on the acceptable premise of the country Zambia having over 95% of the malaria cases caused by one species [44]. However, the reality as demonstrated in this study, is that in the rare case when species other than *P. falciparum* are present, they will be missed and misdiagnosed [28, 45]. It would be prudent for the government of Zambia to consider the use of RDTs that detect all species in health facilities located in border areas.

Apart from species, this study also assessed gametocyte presence in the blood smears collected and examined. Gametocytes are a natural phenomenon where malaria infections ultimately transition from asexual to sexual forms when in a human being [46–48] and are responsible for transmission of malaria from one host to another. This study compared the proportions of patients with gametocytes between the patients resident in the two neighbouring countries compared to those resident in Zambia. The proportions in the former were significantly higher than in the latter. The findings are interpreted with full recognition of the following considerations, that microscopy is limited in detecting gametocytes [49, 50]; only a small fraction of asexual parasites differentiate into gametocytes [51] and only a fraction of infected individuals harbour gametocytes [49, 51]. However, these results are acceptable given the efforts made to reduce bias in slide reading. The study involved up to three slide readers, all blinded to one another, for all the specimens. As such, the results are accurate to the extent of the capacity of the diagnostic tools employed. As others have suggested, it is important to acknowledge the role of gametocytes in malaria transmission [46–48]. This study further extrapolates this notion in perspective of its potential contribution to transmission of the non-*falciparum* species at the border areas of Zambia with the two neighbouring countries. All conditions permitting, this is a possibility as one study suggests that individuals who enter malaria regions can influence epidemiological dynamics [52]. It is necessary to study further the transmission potential that could exist with the presence of species other than *P. falciparum* and their gametocytes, as part of the process for planning and revising of control and elimination efforts.

With regards to severity of anaemia, the proportion of mild or moderate anaemia was higher than that of severe anaemia overall in our study. However, the cases of severe anaemia were higher in patients residing locally in Zambia than those residing in neighbouring countries. The fact that severe anaemia was not significant in the modelling is highlighted, although noting also that the proportions of severe anaemia were higher among patients with *P. falciparum* than among those infected with other species. This does not rule out the fact that severe anaemia still occurs with species other than *P. falciparum* [2, 28]. This study underscores the fact that *P. falciparum* is virulent, and the heavy parasite burden associated with it lends to development of anaemia rapidly causing severe red cell haemolysis due to parasitaemia [53]. This could understandably justify the focus on *P. falciparum* malaria diagnosis in Zambia over the other species. With regards to other species, studies have shown that *P. malariae* is characterized by chronic infections, while *P. ovale* has dormant hypnozoites stages, which may explain the lower haemolysis rate [54]. In spite of the varying pathogenesis of malaria in infections with the different species, these infections still demand attention. This is explained in that they may each require additional and well considered administration of interventions, such as primaquine, an anti-hypnozoite drug [54] in the case of *P. ovale*. Severe anaemia cases were observed in both local participants as well as those from the two neighbouring countries, and in infections involving both *P. falciparum* and other species. It is, therefore, necessary for control programmes to consider studying the extents of anaemia severity that different species may be associated with so as to appropriately manage the infections. Severe anaemia is a recognized proxy for severe malaria and has been used to prioritize management of cases [26]. It is recommended that attention be paid to malaria severity with severe anaemia as a proxy, in the management of imported cases that tend to be more severe than local infections. Although this study determined that severity of malaria with regards to anaemia severity was higher in the local infections compared to the imported ones, the discussion is in perspective of other findings that imported malaria infections are more severe than local infections [55].

Age, gender and country of residence as risk factors for presence of particular malaria species and gametocytes were evaluated. However, only country of residence was significantly related. Although there were more females than males and more children than adults with malaria, these factors were not of significant relationship based on penalized logistic regression modelling. Notwithstanding, it is necessary for a country participating in malaria elimination programmes to consider exploring age and gender, as potential factors at play especially that these have been significant elsewhere [36]. The variation between genders has been shown to be possibly related to occupational exposure for women working in vending jobs at late hours [56, 57]. Others have further alluded to unequal balance of power between men and women in households, which bars women in households to access healthcare [57]. On the other hand, genetic factors have been advanced [58] and may be explored. Age as a factor remains critical as demonstrated in a similar study, which found that children were the most affected by imported malaria, advancing weaker immunity [36]. As such children should continue to be prioritized for treatment and care.

In this study, the country malaria patients resided in, was related to the malaria species they carried. While such information on species per country is available [2] and has served as guide on what species to focus on in control strategies among countries, these findings can also be used as an indicator of potential or active malaria importation.

This study was limited on the following scores: it was conducted outside the peak malaria transmission season in Zambia, which could have underestimated the cases detected; it excluded patients without signs and symptoms of malaria and thereby possibly missing asymptomatic cases; its screening capacity to detect all *Plasmodium* species by RDT was limited to *P. falciparum* specific tests, which could have been improved with RDTs that can detect all species; its capacity to confirm both species and gametocytes was limited to microscopy, which could have yielded a more accurate picture with more sensitive methods; it focussed only on malaria-infected humans and their travel history to confirm imported cases, when further tests to confirm onset of infection and detection of infection in vectors, could have increased accuracy.

## Conclusion

Malaria prevalence in North-Western Province is high, with *P. falciparum* as the predominant species. Importation of *P. ovale* and *P. malariae* is happening in the province through human travel across borders with the two neighbouring countries. This study demonstrates that geographic residence of patients is a major risk factor for malaria species and gametocyte presence. It is relevant in that it highlights the status of local and imported malaria and risk factors that could be used for targeted response. The need for enhanced malaria control with specific focus on border controls to detect and treat, for specific diagnosis and treatment according to species obtaining, for further research in the role of species and gametocytes in imported malaria cannot be overemphasized.

## Data Availability

The datasets used and/or analysed during the current study are available from the corresponding author on reasonable request.

## Abbreviations

P: Probability value
χ^2^: Chi-square test
OR: Odds ratio
CI: Confidence interval
WHO: World Health Organization
spp: Species
NMEC: National Malaria Elimination Centre
E8: Elimination 8
Congo D. R.: Democratic Republic of Congo
RDTs: Rapid diagnostic tests
EDTA: Ethylene Diamine Tetra-acetic Acid
HB: Haemoglobin
pH: Potential of hydrogen
WBCs: White blood cells
Mm: Millimetres
G: Grams
Dl: Decilitre
Coef.: Coefficient
SE coef.: Standard error of coefficient
Df: Degrees of freedom
*E*β: Exponent of coefficient
Inf: Infinity
SPSS: Statistical Package for Social Sciences
UNZAHSREC: University of Zambia Health Sciences Research Ethics Committee
NHRA: National Health Research Authority
